# Connecting the Dots: Potential of Data Integration to Identify Regulatory SNPs in Late-Onset Alzheimer's Disease GWAS Findings

**DOI:** 10.1371/journal.pone.0095152

**Published:** 2014-04-17

**Authors:** Samantha L. Rosenthal, M. Michael Barmada, Xingbin Wang, F. Yesim Demirci, M. Ilyas Kamboh

**Affiliations:** 1 Department of Human Genetics, University of Pittsburgh, Pittsburgh, Pennsylvania, United States of America; 2 Alzheimer's Disease Research Center, University of Pittsburgh, Pittsburgh, Pennsylvania, United States of America; University of Leipzig, Germany

## Abstract

Late-onset Alzheimer's disease (LOAD) is a multifactorial disorder with over twenty loci associated with disease risk. Given the number of genome-wide significant variants that fall outside of coding regions, it is possible that some of these variants alter some function of gene expression rather than tagging coding variants that alter protein structure and/or function. RegulomeDB is a database that annotates regulatory functions of genetic variants. In this study, we utilized RegulomeDB to investigate potential regulatory functions of lead single nucleotide polymorphisms (SNPs) identified in five genome-wide association studies (GWAS) of risk and age-at onset (AAO) of LOAD, as well as SNPs in LD (*r^2^*≥0.80) with the lead GWAS SNPs. Of a total 614 SNPs examined, 394 returned RegulomeDB scores of 1–6. Of those 394 variants, 34 showed strong evidence of regulatory function (RegulomeDB score <3), and only 3 of them were genome-wide significant SNPs (*ZCWPW1*/rs1476679, *CLU*/rs1532278 and *ABCA7/*rs3764650). This study further supports the assumption that some of the non-coding GWAS SNPs are true associations rather than tagged associations and demonstrates the application of RegulomeDB to GWAS data.

## Introduction

Over 1200 genome-wide association studies (GWAS) have been published since 2005 [1]. While some of these studies have been crucial for determining genes responsible for disease phenotypes, including determination of genes involved in inflammatory bowel disease and age-related macular degeneration, the majority of variants identified show modest effect size at best. Furthermore, 88% of significant variants are located in either intronic or intergenic regions that do not encode proteins, suggesting their association with disease may occur for reasons other than changes in protein structure and/or function [2].

Given these findings, researchers recently have begun to deliberate implications of these non-coding variants. One such consideration is the possibility that, splice site variants and promoters aside, introns and intergenic regions are not “junk DNA” as previously believed, but possess regulatory properties which modify gene expression. Indeed, only 2% of the human genome encodes proteins, the remaining 98% is not “functional” in the sense that it does not encode proteins. Rather, the bulk of the genome is comprised of repeat regions, introns, and transposons [3]. Multiple molecular techniques have been employed to determine chromatin structure, methylation, and protein motifs and binding to assess the effect of non-coding variants on transcription [4]. RegulomeDB is a database developed to capture these data, and subsequently, assess the likelihood that a particular variant affects transcription factor binding. The advent of such databases is advantageous for studying gene associations of complex diseases [2].

Late-onset Alzheimer's disease (LOAD) is one such disease that may be better understood by examining the regulatory function of associated SNPs. Thus far, genome-wide association studies (GWAS) of LOAD have identified over 20 significantly associated risk loci [5]–[7]. In addition, several suggestive loci for risk and age-at-onset (AAO) of AD have also been implicated [8], [9]. Of these loci, only one, *APOE*, shows a strong effect size, which substantially increases risk for individuals homozygous for the APOE*4 allele especially after age 75 [10], [11]. The remaining loci have only weak to modest effect sizes. In this study, we have demonstrated the utility of two publicly available bioinformatics tools, Broad Institute's SNP Annotation and Proxy search (SNAP) tool (http://www.broadinstitute.org/mpg/snap/) [12] and RegulomeDB (http://regulomedb.org) [2], to investigate potential regulatory functions of recently identified, non-*APOE* variants (index and proxy SNPs) for known and suggestive loci associated with risk and AAO of LOAD.

## Methods

### SNP selection

We selected a total of 44 genome-wide significant or suggestive single-nucleotide polymorphisms (SNPs) reported for risk or AAO of AD (see **Table S1**). Included among these SNPs were the 28 genome-wide significant SNPs from 21 non-*APOE* LOAD risk loci (*PICALM, BIN1, CD33, CD2AP, MS4A4A/MS4A6E, ABCA7, EPHA1, CLU, CR1, HLA-DRB5/HLA-DRB1, PTK2B, SORL1, SLC24A4/RIN3, DSG2, INPP5D, MEF2C, NME8, ZCWPW1, CELF1, FERMT2*, and *CASS4*) [5]-[7] and 16 SNPs from novel suggestive loci identified in two GWAS of risk and AAO of LOAD (*DCHS, HRK/RNFT2, ADAMTS9, KCNV2/VLDLR, LEMD2/MLN/MIR1275, LOC390958/Sec11C, ZNF592/ALPK3/SLC28A1, PSMD1/HTR2B/ARMC9, NRXN3, PPP1R3B, MMP3/MMP12, FLJ37543, PCDH7, LOC440390, MAPRE1P2* pseudogene, and *PP1R2P5* pseudogene) [8], [9]. IRB approval and informed consent procedures were outlined in each of the publications from which SNPs were selected [5]-[9].

### Linkage Disequilibrium

Following SNP selection, we utilized the SNAP web portal [accessed 4 September 2013] [12] to identify SNPs in linkage disequilibrium (LD) (*r^2^*≥0.80) with our SNPs of interest. SNAP allows users to find proxy SNPs based upon LD determined using the CEU populations from the International HapMap (v3) or 1000 Genomes Pilot 1 projects. SNAP searches were not limited by array and the identified SNPs could include the queried SNPs as proxies for themselves. At *r* ^2^≥0.80, the SNAP portal found 570 SNPs in LD with the 44 GWAS SNPs. SNAP proxy searches were repeated with *r^2^* thresholds of 0.90 and 1.0 to better assess associations among related SNPs. These higher thresholds yielded a total of 472 and 191 identified SNPs, respectively. As expected, the number of identified SNPs in LD with the 44 published SNPs decreased as the *r^2^* threshold increased. **Table 1** summarizes the total number of SNPs in LD at all three thresholds for both HapMap3 and 1000 Genomes searches. All published SNPs and their respective proxy SNPs for each *r^2^* threshold are listed in **Table S1**.

**Table 1:** Number of SNPs in linkage disequilibrium for all published GWAS SNPs for HapMap3 and 1000 Genomes populations at tested *r^2^* thresholds.

	Linkage Disequilibrium (*r^2^*) Threshold		
	0.80	0.90	1.0
**1000 Genomes**	612	466	189
**Hap Map 3**	122	85	62
**TOTAL (overlaps removed)**	614	472	191

### RegulomeDB

RegulomeDB is a database providing functional annotation of SNPs as determined by data from the ENCODE Project Consortium (2012), NCBI Sequence Read Archive, and other sources totaling 962 data sets. It is free and publicly accessible (http://www.regulomedb.org) and has a straight-forward interface. With almost 60 million annotations, this tool will be invaluable for future examination of gene expression and disease traits. Variants can be classified into one of four RegulomeDB categories with scores ranging from 1 to 6 indicating putative functions. Scores and corresponding functional evidence are listed in **Table 2**. All reported SNPs and SNPs in LD (using the *r^2^*≥0.80 list) were examined for potential regulatory functions using RegulomeDB (http://regulomedb.org, accessed [4 September 2013]) [2].

**Table 2:** RegulomeDB category summaries [2].

Category	Description
**Likely to affect binding and linked to expression of a gene target**	
1a	eQTL + TF binding + matched TF motif + matched DNase footprint + DNase peak
1b	eQTL + TF binding + any motif + DNase footprint + DNase peak
1c	eQTL + TF binding + matched TF motif + DNase peak
1d	eQTL + TF binding + any motif + DNase peak
1e	eQTL + TF binding + matched TF motif
1f	eQTL + TF binding/DNase peak
**Likely to affect binding**	
2a	TF binding + matched TF motif + matched DNase footprint + DNase peak
2b	TF binding + any motif + DNase footprint + DNase peak
2c	TF binding + matched TF motif + DNase peak
	
**Less likely to affect binding**	
3a	TF binding + any motif + DNase peak
3b	TF binding + matched TF motif
**Minimal binding evidence**	
4	TF binding + DNase peak
5	TF binding or DNase peak
6	Motif hit

## Results

Of the 614 SNPs examined in RegulomeDB, 220 had RegulomeDB scores of “No Data”, and the remaining 394 returned scores of 1–6. Of those 394 variants, 34 had a RegulomeDB score of less than 3 (**Table 3** and **Table S2**), indicating a relatively high degree of evidence for potential regulatory function (“likely to affect binding”). Interestingly only 3 of these 34 SNPs were the reported genome-wide significant SNPs (*ZCWPW1/*rs1476679, score = 1f; *ABCA7*/rs3764650, score = 2b; and *CLU*/rs1532278, score = 2b), one was a reported suggestive SNP (*HRK/RNFT2*/rs17429217, score = 2b), and the remaining 30 were in LD (*r^2^*≥0.80) with the 44 lead SNPs reported in LOAD GWA studies [5]–[9]. **Table 4** summarizes LD between regulatory SNPs with RegulomeDB score of <3 and published GWAS SNPs. Only one of the 34 SNPs had a score of 1b, while 18 had a score of 1f, 4 returned a score of 2a, and 11 a score of 2b.

**Table 3:** Details of study SNPS with putative regulatory function (RegulomeDB Score <3).

**Coordinate**	**dbSNP ID**	**RegulomeDB**	**Gene/Locus**	**Position** *	**eQTL**	**Motifs**	**Protein**
**(0-based)**		**Score**	**(per dbSNP)**				**Binding**
chr11:59936978	rs667897	1b	*MS4A* region	intergenic	*MS4A4A*	TCF11:MafG	SMARCC2
				downstream *MS4A6*		NFE2L2	STAT1
				downstream *MS4A2*			JUN
							MXI1
							YY1
							CEBPB
							GTF2F1
							FOXA1
							FOS
							USF1
							STAT3
							BRCA1
							EP300
							POLR2A
							ELK4
							PRDM1
							RFX5
							GATA2
							TRIM28
							SETDB1
							JUND
chr11:47461692	rs7103835	1f	*RAPSN*	intron 4	*C1QTNF4*		
chr11:47530023	rs6485758	1f	*CELF1*	intron 1	*C1QTNF4*		
chr11:47572278	rs11039290	1f	*CELF1*	intron 1	*C1QTNF4*	Glis2	
						Mtf1	
chr11:47434985	rs2293576	1f	*SLC39A13*	exon 5	*C1QTNF4*		RAD21
					*MYBPC3*		
**Coordinate**	**dbSNP ID**	**RegulomeDB**	**Gene/Locus**	**Position** *	**eQTL**	**Motifs**	**Protein**
**(0-based)**		**Score**	**(per dbSNP)**				**Binding**
					*SPI1*		
chr11:47509136	rs7933019	1f	*CELF1*	intron 2	*C1QTNF4*		
chr11:47600437	rs2280231	1f	*NDUFS3*	5′ UTR	*C1QTNF4*		BCLAF1
							CHD2
							CREBBP
							CTBP2
							E2F1
							E2F4
							EFL1
							ELK4
							EP300
							ERG
							ETS1
							EWSR1
							FLI1
							GABPA
							GATA1
							GTF2F1
							HNF4A
							IRF1
							IRF4
							JUNB
							JUND
							MYC
							NFKB1
							NR2C2
							PAX5
							POLR2A
							SIX5
**Coordinate**	**dbSNP ID**	**RegulomeDB**	**Gene/Locus**	**Position** *	**eQTL**	**Motifs**	**Protein**
**(0-based)**		**Score**	**(per dbSNP)**				**Binding**
							SMARCB1
							SMARCC1
							SP1
							STAT1
							TAF1
							TBP
							TCF4
							TRIM28
							USF1
							WRNIP1
							ZBTB7A
							ZEB1
chr11:47662931	rs7120548	1f	*MTCH2*	intron 1	*C1QTNF4*		
					*MYBPC3*		
					*SPI1*		
chr11:47811308	rs7114011	1f	*NUP160*	intron 29	*C1QTNF4*	AP-3	
					*MYBPC3*	Oct-1	
					*SPI1*	Irx-3	
chr7:100004445	**rs1476679**	1f	***ZCWPW1***	intron 11	*GATS*		RFX3
					*PILRB*		CTCF
					*TRIM4*		
chr8:27220309	rs17057043	1f	*PTK2B*	intron 5	*DPYSL2*	N-Myc	IRF1
					*PTK2B*	RBP-Jkappa	
chr11:59885119	rs1303615	1f	*MS4A* region	intergenic	*MS4A4A*		
				downstream of *MS4A2*			
				downstream *MS4A6A*			
chr11:59936756	rs617135	1f	*MS4A* region	intergenic	*MS4A4A*		POLR2A
				downstream *MS4A6A*			MAX
**Coordinate**	**dbSNP ID**	**RegulomeDB**	**Gene/Locus**	**Position** *	**eQTL**	**Motifs**	**Protein**
**(0-based)**		**Score**	**(per dbSNP)**				**Binding**
							SMARCC2
chr11:59961485	rs11230180	1f	*MS4A* region	intergenic	*MS4A4A*		JUNB
				upstream *MS4A6A*			NKB1
				downstream *MS4A4E*			
chr11:59966294	rs2123314	1f	*MS4A* region	intergenic	*MS4A4A*		
				upstream *MS4A6A*			
				downstream *MS4A4E*			
chr11:59989429	rs2081547	1f	*MS4A4E*	intron 2	*MS4A4A*		CEBPB
							JUN
							JUND
chr11:60013856	rs655231	1f	*MS4A* region	intergenic	*MS4A4A*		RFX3
				upstream *MS4A4E*			
				upstream *MS4A4A*			
chr15:85425096	rs12917429	1f	*SLC28A1*	intergenic	*NMB*		
				upstream *SLC28A1*			
chr15:85429355	rs12909280	1f	*SLC28A1*	intron 1	*NMB*		RFX3
chr11:60019149	rs636317	2a	*MS4A* region	intergenic	*-------*	TAL1	CTCF
				upstream *MS4A4E*		CTCF	RAD21
				upstream *MS4A4A*			FOXA1
							SMC3
							BCLAF1
							YY1
							POU2F2
							ZNF143
chr11:60019160	rs636341	2a	*MS4A* region	intergenic	*-------*	CTCF	CTCF
				upstream *MS4A4E*		STAT1:STAT1	RAD21
				upstream *MS4A4A*		C/EBPbeta	FOXA1
							SMC3
**Coordinate**	**dbSNP ID**	**RegulomeDB**	**Gene/Locus**	**Position** *	**eQTL**	**Motifs**	**Protein**
**(0-based)**		**Score**	**(per dbSNP)**				**Binding**
							BCLAF1
							YY1
							POU2F2
							ZNF143
chr11:85815029	rs1237999	2a	*PICALM* region	intergenic	*-------*	AP-1	JUN
				upstream *PICALM*		Jundm2	JUNB
							JUND
							FOS
chr19:1046519	**rs3764650**	2a	***ABCA7***	intron 13	*-------*	TBX22	SP1
						TBX18	HNF4A
						TBX15	HNF4G
						HNF4alpha1	BHLHE40
						COUPTF	USF1
						HNF4	USF2
						COUP-TF = HNF-4	
						NR2F1	
chr12:117295332	**rs17429217**	2b	***HRK/RNFT2*** ** region**	intergenic	*-------*	HNF4 = COUP	EBF1
				downstream *RNFT*		Hnf4a	
				downstream *HRK*			
chr6:47447040	rs4715019	2b	*CD2AP*	intron 1	*-------*	Irx-3	POLR2A
						Sox15	
						HoxB5	
						Zfp105	
						Hoxa3	
						DIx1	
						Hoxb8	
						Irx6	
						Hoxa6	
**Coordinate**	**dbSNP ID**	**RegulomeDB**	**Gene/Locus**	**Position** *	**eQTL**	**Motifs**	**Protein**
**(0-based)**		**Score**	**(per dbSNP)**				**Binding**
						Hoxb6	
						Hoxb5	
chr8:27466314	**rs1532278**	2b	***CLU***	intron 3	*-------*	Nkx2-5	NANOG
							TAF1
							USF1
							MAX
							USF2
							GATA2
chr11:59936925	rs7933202	2b	*MS4A* region	intergenic	*-------*	DMRT3	POLR2A
				downstream *MS4A6A*			SMARCC2
							STAT1
							JUN
							YY1
							CEBPB
							GTF2F1
							FOS
							USF1
							STAT3
							EP300
							ELK4
							MXI1
							FOXA1
							BRCA1
							PRDM1
							GATA2
							TRIM28
							SETDB1
							JUND
chr11:85811237	rs542126	2b	*PICALM* region	intergenic	*-------*	E47	HNF4A
**Coordinate**	**dbSNP ID**	**RegulomeDB**	**Gene/Locus**	**Position** *	**eQTL**	**Motifs**	**Protein**
**(0-based)**		**Score**	**(per dbSNP)**				**Binding**
				upstream *PICALM*			RXRA
							POLR3A
							USF2
							USF1
chr19:1047686	rs4147911	2b	*ABCA7*	intron 16	*-------*	HEN1	IKZF1
chr20:54997567	rs6024870	2b	*CASS4*	intron 2	*-------*	Pax-3	TCF4
							CTCF
							FOS
							RAD21
chr2:127888336	rs11689287	2b	*BIN1* region	intergenic	*-------*	FOXL1	CTCF
				upstream *BIN1*		Oct-1	
						Six6	
						FOXP1	
						Tbp	
chr3:64918621	rs812651	2b	*ADAMTS9-AS2*	intron 4	-------	RBP-Jkappa	HNF4A
							SETDB1
chr8:27219986	rs73223431	2b	*PTK2B*	intron 5	*-------*	TRUE	MYC
						TBX15	GATA1
						TBX18	CDX2
						TBX22	POLR2A
						T	JUN
						Brachyury	NKFB1
							HNF4A
							GATA2
chr8:27468502	rs867230	2b	*CLU*	intron 1	*-------*	MEF-2	GATA1
						Zfp740	GABPA

Bolded SNPs are published GWAS SNPs.

*Upstream/downstream designation based upon gene direction per NCBI.

**Table 4:** Linkage disequilibrium for published GWAS SNPs with functional proxies (RegulomeDB score <3) according to SNAP search.

GWAS SNP	Functional Proxy SNP	RegulomeDB Score
*ABCA7*/rs3764650	***ABCA7*** /rs3764650***	2a
	*ABCA7*/rs4147911**	2b
*HRK/RNFT2*/rs17429217	***HRK/RNFT2*** /rs17429217***	2b
*ADAMTS9*/rs704454	*ADAMTS9-AS2*/rs812651**	2b
*CASS4*/rs7274581	*CASS4*/rs6024870*	2b
*ZCWPW1*/rs1476679	***ZCWPW1*** /rs1476679***	1f
*CD2AP*/rs9296559	*CD2AP*/rs4715019***	2b
*CD2AP*/rs9349407	*CD2AP*/rs4715019***	2b
*CELF1*/rs10838725	*SLC39A13*/rs2293576*	1f
	*CELF1*/rs7933019**	1f
	*NDUFS3*/rs2280231**	1f
	*MTCH2*/rs7120548*	1f
	*NUP160*/rs7114011**	1f
	*CELF1*/rs11039290**	1f
	*CELF1*/rs6485758**	1f
	*RAPSN*/rs7103835*	1f
*PTK2B*/rs28834970	*PTK2B*/rs17057043*	1f
	*PTK2B*/rs73223431*	2b
*CLU*/rs1532278	***CLU*** /rs1532278***	2b
	*CLU*/rs867230*	2b
*PICALM*/rs561655	*PICALM*/rs1237999*	2a
	*PICALM*/rs542126*	2b
*ZNF592/ALPK3/SLC28A1*/rs3743162	*SLC28A1*/rs12917429***	1f
	*SLC28A1*/rs12909280***	1f
*BIN1*/rs7561528	*BIN1*/rs11689287**	2b

GWAS SNP	Functional Proxy SNP	RegulomeDB Score
*MS4A4A*/rs4938933	*MS4A* region/rs11230180*	1f
	*MS4A* region/rs2123314*	1f
	*MS4A4E*/rs2081547*	1f
	*MS4A*region/rs655231**	1f
	*MS4A* region/rs636341**	2a
	*MS4A* region/rs636317**	2a
	*MS4A* region/rs7933202*	2b
*MS4A6A*/rs610932	*MS4A* region/rs7933202*	2b
	*MS4A* region/rs667897*	1b
	*MS4A* region/rs1303615*	1f
	*MS4A* region/rs617135*	1f

Linkage disequilibrium (*r^2^*) values are indicated as—*≥0.80, **≥0.90, and *** = 1.0.

Bolded SNPs are GWAS SNPs with regulatory function.

A total of 10 confirmed loci and 3 suggestive loci harbored SNPs with a RegulomeDB score <3. The SNP with the most evidence for regulatory function was rs667897 with a RegulomeDB score of 1b. This SNP is an intergenic SNP located in the *MS4A* region, just downstream of *MS4A6A*. Nine other SNPs in the *MS4A* region (of the 157 SNPs tested in this region) also had scores of less than 3, as well as 20 SNPs in 9 other confirmed LOAD risk loci: *ZCWPW1* (1 of 8 SNPs tested), *CLU* (2 of 10 SNPs tested), *ABCA7* (2 of 7 SNPs tested), *CELF1* (8 of 25 SNPs tested), *PTK2B* (2 of 6 SNPs tested), *CASS4* (1 of 11 SNPs tested), *PICALM* (2 of 93 SNPs tested), *CD2AP* (1 of 69 SNPs tested), and *BIN1* (1 of 6 SNPs tested). Remarkably, eight SNPs in the *CELF1* gene region on chromosome 11 (*SLC39A13*/rs2293576, *CELF1*/rs7933019, *NDUFS3*/rs2280231, *MTCH2*/rs7120548, *NUP160*/rs7114011, *CELF1*/rs11039290, *CELF1*/rs6485758, and *RAPSN*/rs7103835) with scores of 1f are in LD with the genome-wide significant *CELF1*/rs10838725 SNP which by itself is not functional according to RegulomeDB (score = 6). All eight are eQTLs for *C1QTNF4*, and three of them (*SLC39A13*/rs2293576, *MTCH2*/rs7120548, and *NUP160*/rs7114011) also affect expression of *MYBPC3* and *SPI1*. Three other suggestive novel loci, *ADAMTS9, ZNF592/ALPK3/SLC28A1*, and *HRK/RNFT2*, also had variants with strong evidence for regulatory function with scores of 2b (1 of 6 SNPs tested), 1f (2 of 7 SNPs tested), and 2b (1 of 1 SNP tested), respectively.

Of the 30 SNPs that were in LD with reported genome-wide significant variants and had high evidence of regulatory function, 10 were located in the *MS4A* region, including the SNP with the most evidence for regulatory function, rs667897 (RegulomeDB score = 1b). RegulomeDB cites rs667897 affects binding of 21 different proteins including BRCA1, SMARCC2, FOXA1, JUN, and POLR2A and falls within both TCF11:MafG and NFE2L2 binding motifs. Six other SNPs in the *MS4A* region, including 5 intergenic (rs1303615, rs617135, rs11230180, rs2123314, and rs655231) and 1 intronic (*MS4A4E*/rs2081547) SNPs, had RegulomeDB scores of 1f, and similar to the top hit rs667897, all are eQTLs for *MS4A4A* as evidenced by work in monocytes. Some of the protein binding affected by these SNPs include CEBPB, JUN, JUND, POLR2A, and SMARCC2. These are the same proteins that are also affected by top *MS4A* region hit, rs667897, however, motifs containing these variants have yet to be determined. Three more SNPs in the *MS4A* region, rs636317, rs636341, and rs7933202 were likely to affect binding according to RegulomeDB (score  = 2a, 2a, and 2b, respectively).

The reported *ZCWPW1*/rs1476679 SNP (RegulomeDB score = 1f) is an eQTL for *GATS, PILRB*, and *TRIM4*, and similar to other functional variants in our dataset, affects binding of RFX3, as well as CTCF. Two intronic *CLU* SNPs, rs1532278 and rs867230, show evidence of regulatory function (RegulomeDB score = 2b, each). The genome-wide significant *CLU*/rs1532278 SNP, located in intron 3 of *CLU* affects binding of NANOG, TAF1, USF1, MAX, USF2, and GATA2 and is situated in the Nkx2-5 binding motif. The second *CLU*/rs867230 variant, located in the first intron of *CLU*, affects binding of GATA1 and GABPA and alters the MEF-2 and Zfp740 motifs.

A genome-wide significant *ABCA7*/rs3764650 SNP (RegulomeDB score = 2a), has indications for binding six different proteins (SP1, HNF4A, HNF4G, BHLHE40, USF1, and USF2) and is located in the binding motifs for TBX22, TBX18, TBX15, HNF4alpha1, COUPTF, HNF4, COUP-TF = HNF-4, NR2F1. Another *ABCA7*/rs4147911 SNP (RegulomeDB score = 2b) affects binding of IKZF1.

An eQTL for both *DPYSL2* and *PTK2B*, rs17057043 (RegulomeDB score = 1f) is located in intron 5 of *PTK2B*, which is part of the N-Myc and RBP-Jkappa binding motifs, and affects binding of IRF1. *PTK2B*/rs73223431 SNP, also located in intron 5 of *PTK2B*, has a score of 2b. Similar to *ABCA7*/rs3764650 (RegulomeDB score = 2b), *PTK2B*/rs73223431 falls in binding motifs of TBX22, TBX15, and TBX18, among others.

## Discussion

As the list of associated LOAD risk loci continues to grow, it becomes increasingly important to decipher the biological underpinnings of these associations. If we accept that these associations are real, we must endeavor to explain them. Since many of these risk variants are non-coding, one logical explanation for their association is an effect on gene expression. The ENCODE project has provided invaluable contributions to this area of research with a wealth of data that is publicly available for interpretation and expansion. These data are ideal for generating hypotheses and furthering our understanding of gene expression and epistasis. Here we have used two publicly available bioinformatics tools, SNAP tool and RegulomeDB, to investigate potential regulatory functions of non-*APOE* SNPs implicated with risk and AAO of LOAD.

Of the 21 non-*APOE* genome-wide significant risk loci, ten— *ZCWPW1, CLU*, *ABCA7, MS4A4A/MS4A6E*, *PICALM, CD2AP, BIN1*, *CELF1, CASS4*, and *PTK2B*—had SNPs with functional evidence. Of the 16 suggestive novel loci, three-- *HRK/RNFT2*, *ADAMTS9*, and *ZNF592/ALPK3/SLC28A1--* had SNPs with functional evidence. Importantly, only three of the 34 SNPs with evidence for potential regulatory function based on RegulomeDB score were the reported genome-wide significant SNPs [*ZCWPW1*/rs1476679 (score = 1f), *CLU*/rs1532278 (score = 2b), and *ABCA7*/rs3764650 (score = 2b)] and one was a reported suggestive SNP [*HRK/RNFT2*/rs17429217 (score = 2b)]. All three reported genome-wide significant SNPs are intronic and our findings suggest that they, rather than the SNPs in LD with them, are causative for LOAD risk via a regulatory mechanism.

None of the ten *MS4A* region SNPs with a score of <3 are reported GWAS SNPs, indicating the difficulty of differentiating between a true signal and a tag signal in association studies, as well as highlighting the complexity of interactions between genetic variants and disease risk. Of the remaining 24 putative regulatory variants representing 12 loci other than the *MS4A* region, we observe some thought-provoking outcomes. For example, synonymous variant *SLC39A13*/rs2293576 (in LD with *CELF1*/rs10838725, *r^2^*≥0.8) is unique in this dataset because it is the only SNP with regulatory evidence that resides in an exon, reminding us that regulatory elements can be found within coding sequences as well as in intergenic regions and introns. Furthermore, eight putative regulatory variants located in six different genes (including the synonymous *SLC39A13*/rs2293576 variant) are in LD with the reported *CELF1*/rs10838725 SNP, and all are eQTLs for *C1QTNF4*. These results suggest future work should examine *C1QTNF4* (aka *CTRP4*) as a potential player in LOAD risk in addition to currently implicated *CELF1* gene. *C1QTNF4* is an inflammatory cytokine capable of activating both Stat3/IL6 and NF-κB pathways, as shown in cancer cells [13]. The implication of the inflammatory pathway in AD pathogenesis and the inverse association between AD and cancer may explain in part the observed relationship between these SNPs and their effect on *C1QTNF4* expression [14], [15].

According to RegulomeDB, the binding of the IKAROS family zinc finger 1 (Ikaros) transcription factor, IKZF1, is affected by *ABCA7/*rs4147911 (score = 2b). It is worth noting that the expression of another LOAD risk gene, *INPP5D*, is regulated by the Ikaros transcription factor family in B cells [16], suggesting a potential functional link between *ABCA7* and *INPP5D*. Similarly, RegulomeDB findings suggest other proteins whose binding seems to be affected by variants at different LOAD loci (**Table 3**). Another position of interest is intron 5 of *PTK2B*. Two variants in this intron had RegulomeDB scores less than 3 (rs17057043, score = 1f and rs73223431, score = 2b), suggesting that intron 5 of *PTK2B* may play an important role in affecting the binding of regulatory proteins and consequently the risk of LOAD.

Variants in reported suggestive novel loci for AAO of AD, *ZNF592/ALPK3/SLC28A1*, *HRK/RNFT2*, and *ADAMTS9*, are also of functional importance as reflected by RegulomeDB scores of 1f, 2b, and 2b, respectively. Both rs12917429 and rs12909280 in the *SLC28A1* region are eQTLs for *neuromedin B* (*NMB*), with the latter SNP suggested to affect binding of RFX3. According to GeneCards [17] NMB is a ligand that binds to bombesin receptors to instigate smooth muscle contractions. The bombesin peptides and receptors have been implicated in a variety of cellular processes and are frequently overexpressed in cancer cells [18], [19]. RFX3 has been shown to be responsible for proper Corpus Callosum development in mice [20]. RFX3 also affects expression of glucokinase and subsequently affects differentiation and function of beta cells [21]. Two other SNPs with RegulomeDB scores of 1f, *ZCWPW1*/rs1476679 and rs655231 (*MS4A* region), show indications for affecting binding of RFX3 in K562 (chronic myelogenous leukemia, CML) cells. Given the proposed link between insulin resistance and AD as a result of insulin degrading enzyme (IDE), RFX3 may be an interesting transcription factor to examine in the context of LOAD pathogenesis [22].

Although RegulomeDB is an extensive database for the annotation of variants' effects on gene expression, it provides information for only selected DNA binding elements in certain cell types. A total of 220 variants of the 614 we examined returned scores of “No data,” meaning we cannot argue against their involvement in gene expression as related to LOAD pathogenesis. Along the same lines, some loci have a markedly higher number of SNPs that have been tested for expression effects than others. Thus, we make no assumptions that the mere number of putative regulatory variants for a given locus is indicative of the magnitude of that locus' role in risk and disease process. Moreover, the primary focus of our study was RegulomeDB and prediction of regulatory effects on gene expression based on the data included in that database. Therefore some other regulatory mechanisms, such as regulation of RNA splicing, or prediction of changes in protein structure and/or function were not covered as part of this study.

In conclusion, these results highlight a number of important considerations for the interpretation of future GWAS data including the necessity of carefully examining LD structure for SNPs showing association with disease risk. Additionally, careful attention should be paid to the regulatory function of associated SNPs and those in LD with such SNPs to clarify correlation between disease and associated variants and better understand complex disease mechanisms. These factors will be critical for elucidating genetic mechanisms that are truly causal for LOAD. Although the cellular pathology of the disease appears to be more widely agreed upon, the molecular basis is still elusive, requiring resolution before pathogenesis is completely comprehended. Identification of potential therapeutic targets can be expedited with a more extensive molecular understanding of the disease process. Given the replication of association of loci with unclear or unknown functions with LOAD risk, future studies should aim to determine their functions both in normal and disease states to identify their roles in disease pathogenesis.

## Supporting Information

Table S1 **Proxy SNPs from SNAP search (HapMap3 and 1000 Genomes combined) for published GWAS SNPs.**
(PDF)

Table S2 **RegulomeDB Scores and Coordinates for all associated SNPs and SNPs in LD** ***r^2^*** **≥0.8.**
(PDF)
