# Supplementary material for: Connecting the Dots: Potential of Data Integration to Identify Regulatory SNPs in Late-Onset Alzheimer's Disease GWAS Findings
Source: PLoS One. 2014 Apr 17;9(4):e95152. doi: 10.1371/journal.pone.0095152 (PMC3990600; doi:10.1371/journal.pone.0095152)
Supplement: Table S1 — Proxy SNPs from SNAP search (HapMap3 and 1000 Genomes combined) for published GWAS SNPs. (PDF) [file pone.0095152.s001.pdf]

Table S1. Proxy SNPs from SNAP search (HapMap3 and 1000 Genomes combined) for published GWAS SNPs.

SNP (Nearest Gene)	Proxy		
	r ^{2} **≥ 0.8**	***r*** ^{2} **≥ 0.9**	***r*** ^{2} **= 1.0**
**rs1037757**[1,2,5,6]			
LOC390958/Sec11C			
**rs10517270**[1,2]	**rs10517270**	**rs10517270**	**rs10517270**
MAPRE1P2 **(pseudogene)**			
**rs11738335**	**rs11738335**	**rs11738335**	**rs11738335**
FLJ37543	rs7714841	rs7714841	rs7714841
	rs11750531	rs11750531	rs11750531
	rs16893320	rs16893320	rs16893320
	rs7728181	rs7728181	rs7728181
	rs16893322	rs16893322	rs16893322
	rs35438183	rs35438183	rs35438183
	rs6899064	rs6899064	rs6899064
	rs13175381	rs13175381	rs13175381
	rs6883938	rs6883938	rs6883938
	rs4700424	rs4700424	rs4700424
	rs6880041	rs6880041	
	rs35673179	rs35673179	
	rs11746049	rs11746049	
	rs11750644	rs11750644	
	rs16893553	rs16893553	
	rs6896690	rs6896690	
	rs1501845	rs1501845	
	rs13156487	rs13156487	
	rs11739353	rs11739353	
	rs13175459	rs13175459	
	rs11743702	rs11743702	
	rs11747263	rs11747263	
	rs13186555	rs13186555	

	rs13163936	rs13163936	
	rs4700430	rs4700430	
	rs58408393	rs58408393	
	rs12517109	rs12517109	
	rs12520360	rs12520360	
	rs13156637	rs13156637	
	rs4699979	rs4699979	
	rs4699980	rs4699980	
	rs4700432	rs4700432	
	rs17429209	rs17429209	
	rs13170002	rs13170002	
	rs116432267	rs116432267	
	rs13160822	rs13160822	
	rs12519720	rs12519720	
	rs12519709	rs12519709	
	rs12522405	rs12522405	
	rs6872706	rs6872706	
	rs1123763	rs1123763	
	rs1123764	rs1123764	
	rs12521924	rs12521924	
	rs13159935	rs13159935	
	rs12514378	rs12514378	
	rs11738462	rs11738462	
	rs7704929		
	rs6870236		
	rs13154578		
	rs11740126		
	rs11747208		
**rs11767557**[2,3]	**rs11767557**	**rs11767557**	**rs11767557**
EPHA1	rs11763230	rs11763230	rs11763230
	rs11762262	rs11762262	rs11762262
	rs56402156	rs56402156	rs56402156
	rs10808026	rs10808026	

	rs7791765	rs7791765	
**rs12808148**	**rs12808148**	**rs12808148**	**rs12808148**
MMP3/MMP12			
**rs12933233**	**rs12933233**	**rs12933233**	**rs12933233**
LOC440390			
**rs1357692**	**rs1357692**	**rs1357692**	**rs1357692**
***PPP1R2P5* (pseudogene)**	rs1357694	rs2030675	
	rs2030674	rs2030677	
	rs1916921	rs2971894	
	rs2030675		
	rs2030677		
	rs2971650		
	rs2971894		
	rs1524288		
	rs2971892		
	rs7559405		
	rs13017797		
	rs1916920		
**rs1466662**[1,2]	**rs1466662**	**rs1466662**	**rs1466662**
DCHS2	rs1466661	rs1466661	rs1466661
	rs35648280	rs35648280	rs35648280
	rs17373494	rs17373494	rs17373494
	rs62331930	rs62331930	rs62331930
	rs35190023	rs35190023	rs35190023
	rs57866349	rs57866349	rs57866349
	rs17301482	rs17301482	rs17301482
	rs12500118	rs12500118	rs12500118
	rs4696572	rs4696572	rs4696572
	rs9790842	rs9790842	
	rs17373598		

	rs62330361		
	rs990185		
	rs1490674		
	rs10857275		
	rs1844672		
	rs2130714		
	rs9307922		
	rs10517591		
	rs13150461		
**rs1532278**	**rs1532278**	**rs1532278**	**rs1532278**
CLU	rs1532276	rs1532276	
	rs11787077	rs11787077	
	rs1532277	rs1532277	
	rs4236673	rs4236673	
	rs9331896	rs9331896	
	rs11136000	rs11136000	
	rs7982		
	rs2070926		
	rs867230		
**rs17429217**[1,2,5,6]	**rs17429217**	**rs17429217**	**rs17429217**
HRK/RNFT2			
**rs17764668**	**rs17764668**	**rs17764668**	**rs17764668**
NRXN3	rs11628403	rs11628403	
	rs72698407	rs72698407	
	rs11628399	rs11628399	
	rs12586501	rs12586501	
	rs72698405	rs72698405	
	rs12590550	rs12590550	
	rs766649	rs766649	
	rs8021767	rs8021767	
	rs72696796	rs72696796	

	rs12589656	rs12589656	
	rs7158292	rs7158292	
	rs17764518	rs17764518	
	rs17174723	rs17174723	
	rs12586263	rs12586263	
	rs12588956		
**rs2034764**	**rs2034764**	**rs2034764**	**rs2034764**
KCNV2/VLDLR	rs2034763	rs2034763	
	rs1006698	rs1006698	
	rs1026355	rs1026355	
	rs7048820		
	rs10733413		
**rs2104362**	**rs2104362**	**rs2104362**	**rs2104362**
LEMD2/MLN/MIR1275	rs4713697	rs4713697	rs4713697
	rs1555685	rs1555685	rs1555685
	rs9394174	rs9394174	rs9394174
	rs3998107	rs3998107	rs3998107
	rs1555687	rs1555687	rs1555687
	rs1535937	rs1535937	
	rs1555689	rs1555689	
**rs3743162**	**rs3743162**	**rs3743162**	**rs3743162**
ZNF592/ALPK3/SLC28A1	rs3743161	rs3743161	rs3743161
	rs34886124	rs34886124	rs34886124
	rs12909280	rs12909280	rs12909280
	rs12917429	rs12917429	rs12917429
	rs12442557	rs12442557	rs12442557
	rs34130645		
**rs3748140**[6,7]	**rs378140**	**rs378140**	**rs378140**
PPP1R3B			

**rs3752246**[1,2]	**rs3752246**	**rs3752246**	**rs3752246**
ABCA7			
**rs3764650**	**rs3764650**	**rs3764650**	**rs3764650**
ABCA7	rs4147910	rs4147910	
	rs4147911	rs4147911	
	rs73505217	rs73505217	
	rs78410552	rs78410552	
	rs76348507	rs76348507	
**rs3818361**	**rs3818361**	**rs3818361**	**rs3818361**
CR1	rs6701713	rs6701713	rs6701713
	rs2093761	rs2093761	rs2093761
	rs2093760	rs2093760	
	rs10863420	rs10863420	
	rs1830763	rs1830763	
	rs1408078	rs1408078	
	rs4844610	rs4844610	
	rs1408077	rs1408077	
	rs111958034	rs111958034	
	rs2296160	rs2296160	
	rs10863418	rs10863418	
	rs10779336	rs10779336	
	rs6661489	rs6661489	
	rs4562624	rs4844600	
	rs6656401		
	rs4266886		
	rs4844600		
**rs3865444**	**rs3865444**	**rs3865444**	**rs3865444**
CD33	rs33978622	rs33978622	
	rs7245846	rs7245846	
	rs34813869	rs34813869	
	rs1354106		

**rs4938933**	**rs4938933**	**rs4938933**	**rs4938933**
MS4A4A	rs7108663	rs7108663	rs7108663
	rs4938931	rs4938931	rs4938931
	rs1562990	rs1562990	rs1562990
	rs2162254	rs2162254	
	rs4938932	rs4938932	
	rs7930318	rs7930318	
	rs10736701	rs10736701	
	rs10736700	rs10736700	
	rs7128450	rs7128450	
	rs1026252	rs1026252	
	rs1026253	rs1026253	
	rs1026254	rs1026254	
	rs1026255	rs1026255	
	rs78057299	rs78057299	
	rs1530914	rs1530914	
	rs6591559	rs6591559	
	rs7121656	rs7121656	
	rs74452343	rs74452343	
	rs672399	rs672399	
	rs636341	rs636341	
	rs636317	rs636317	
	rs655231	rs655231	
	rs654415	rs654415	
	rs600064	rs600064	
	rs603648	rs603648	
	rs611418	rs611418	
	rs1019671	rs1019671	
	rs1426250	rs1426250	
	rs2015475	rs2015475	
	rs11601689	rs11601689	
	rs1349667	rs1349667	
	rs11826180	rs11826180	

rs2081547	rs2081547
rs4492839	rs4492839
rs1820428	rs1820428
rs56273223	rs56273223
rs11603507	rs11603507
rs4939320	rs4939320
rs11230194	rs11230194
rs7932740	rs7932740
rs7928895	rs7928895
rs7939882	rs7939882
rs72920867	
rs11230201	
rs56357056	
rs7929589	
rs7933805	
rs2123314	
rs11230184	
rs11230183	
rs7116190	
rs7926354	
rs7926344	
rs7926729	
rs11230180	
rs10897011	
rs1834550	
rs2081545	
rs1426253	
rs764859	
rs718376	
rs7936120	
rs17602572	
rs12453	
rs7946992	
rs72918674	

	rs7935829		
	rs7107627		
	rs1426249		
	rs7933202		
	rs7926954		
	rs11605427		
**rs561655**	**rs561655**	**rs561655**	**rs561655**
PICALM	rs59097112	rs59097112	rs59097112
	rs474479	rs474479	
	rs567075	rs567075	
	rs526904		
	rs497816		
	rs1237999		
	rs7110631		
	rs7941541		
	rs631639		
	rs480781		
	rs565719		
	rs541458		
	rs536841		
	rs676733		
	rs10792832		
	rs3851179		
	rs542126		
	rs867611		
	rs493254		
	rs694011		
	rs561646		
	rs682928		
	rs609903		
	rs636355		
	rs602222		
	rs645299		

	rs587038		
	rs673751		
	rs573167		
	rs586274		
	rs543293		
	rs677909		
**rs610932**	**rs610932**	**rs610932**	**rs610932**
MS4A6A	rs634475	rs634475	
	rs662196	rs662196	
	rs631853	rs631853	
	rs624663	rs624663	
	rs583791	rs583791	
	rs632185	rs632185	
	rs107903	rs107903	
	rs652354	rs652354	
	rs684961	rs684961	
	rs606588	rs606588	
	rs617916	rs617916	
	rs558375	rs558375	
	rs583296	rs583296	
	rs595481	rs595481	
	rs493692	rs493692	
	rs534273	rs534273	
	rs510518	rs510518	
	rs504272	rs504272	
	rs525794	rs525794	
	rs1303621	rs1303621	
	rs564912	rs564912	
	rs1303615	rs1303615	
	rs581133	rs581133	
	rs574798	rs574798	
	rs574695	rs574695	
	rs540170	rs540170	

	rs563803	rs563803	
	rs514266	rs514266	
	rs555635	rs555635	
	rs512495	rs512495	
	rs487997	rs487997	
	rs521952	rs521952	
	rs580817	rs580817	
	rs574704	rs574704	
	rs502419	rs502419	
	rs502581	rs502581	
	rs636147	rs636147	
	rs476722	rs476722	
	rs602396	rs602396	
	rs688030	rs688030	
	rs569046	rs569046	
	rs483629	rs483629	
	rs516478	rs516478	
	rs556917	rs556917	
	rs1441586	rs1441586	
	rs667897		
	rs617135		
	rs2278867		
	rs7933202		
	rs7926954		
	rs11605427		
	rs580064		
	rs7935829		
	rs72918674		
	rs7946992		
	rs12453		
	rs17602572		
**rs670139**	**rs670139**	**rs670139**	**rs670139**
MS4A4E	rs666555	rs666555	rs666555

	rs599862	rs599862	rs599862
	rs588084	rs588084	rs588084
	rs652303	rs652303	rs652303
	rs668287	rs668287	rs668287
	rs604085	rs604085	rs604085
	rs1285231	rs1285231	rs1285231
	rs670854	rs670854	rs670854
	rs688460	rs688460	rs688460
	rs584469	rs584469	rs584469
	rs612738	rs612738	rs612738
	rs620612	rs620612	rs620612
	rs673996	rs673996	rs673996
	rs600550	rs600550	rs600550
	rs676309	rs676309	rs676309
	rs678384	rs678384	rs678384
	rs621965	rs621965	rs621965
	rs650943	rs650943	rs650943
	rs1530915	rs1530915	rs1530915
	rs1786140	rs1786140	rs1786140
	rs474123	rs474123	rs474123
	rs657928	rs657928	
	rs627081	rs627081	
	rs664034	rs664034	
	rs592894	rs592894	
	rs4939328	rs4939328	
	rs4939329	rs4939329	
	rs603568	rs603568	
	rs673141	rs673141	
	rs633463	rs633463	
	rs675655	rs675655	
	rs1365247		
	rs12221613		
**rs6701713**	**rs6701713**	**rs6701713**	**rs6701713**

CR1	rs2093761	rs2093761	rs2093761
	rs3818361	rs3818361	rs3818361
	rs2093760	rs2093760	
	rs10863420	rs10863420	
	rs1830763	rs1830763	
	rs1408078	rs1408078	
	rs4844610	rs4844610	
	rs1408077	rs1408077	
	rs111958034	rs111958034	
	rs2296160	rs2296160	
	rs10863418	rs10863418	
	rs10779336	rs10779336	
	rs6661489	rs6661489	
	rs4562624	rs484460	
	rs6656401		
	rs4266886		
	rs4844600		
**rs6856768**	**rs6856768**	**rs6856768**	**rs6856768**
PCDH7	rs35942547	rs35942547	rs35942547
	rs16867949	rs16867949	rs16867949
	rs1980054	rs1980054	rs1980054
	rs2175560	rs4692459	
	rs4692459	rs6847958	
	rs16867945		
	rs6847958		
	rs16867940		
**rs704454**	**rs704454**	**rs704454**	**rs704454**
ADAMTS9	rs704457	rs704457	rs704457
	rs704453	rs704453	rs704453
	rs704456	rs704456	
	rs812651	rs812651	
	rs807411		

**rs744373**	**rs744373**	**rs744373**	**rs744373**
BIN1	rs730482	rs730482	rs730482
**rs753855**	**rs753855**	**rs753855**	**rs753855**
PSMD1/HTR2B/ARMC9			
**rs7561528**	**rs7561528**	**rs7561528**	**rs7561528**
BIN1	rs11680911	rs11680911	
	rs11689287		
	rs35114168		
**rs9296559**	**rs9296559**	**rs9296559**	**rs9296559**
CD2AP	rs9296558	rs9296558	rs9296558
	rs9349407	rs9349407	rs9349407
	rs9473119	rs9473119	rs9473119
	rs9367279	rs9367279	rs9367279
	rs4715019	rs4715019	rs4715019
	rs1004173	rs1004173	rs1004173
	rs7738044	rs7738044	rs7738044
	rs13211285	rs13211285	rs13211285
	rs4711880	rs4711880	rs4711880
	rs1872505	rs1872505	rs1872505
	rs9473126	rs9473126	rs9473126
	rs4715025	rs4715025	rs4715025
	rs9369716	rs9369716	rs9369716
	rs10948361	rs10948361	
	rs9381564	rs9381564	
	rs6931478	rs6931478	
	rs1931837	rs1931837	
	rs4715018	rs4715018	
	rs9369695	rs9369695	
	rs9473117	rs9473117	
	rs9381562	rs9381562	

rs7740963	rs7740963
rs1931833	rs1931833
rs7749271	rs7749271
rs7767350	rs7767350
rs10948363	rs10948363
rs9296561	rs9296561
rs7749167	rs7749167
rs9296564	rs9296564
rs7754282	rs7754282
rs6904764	rs6904764
rs9349413	rs9349413
rs74607583	rs74607583
rs2151974	rs2151974
rs2151975	rs2151975
rs2171089	rs2171089
rs9367284	rs9367284
rs9381575	rs9381575
rs9395279	rs9395279
rs9357546	rs9357546
rs9395283	rs9395283
rs9349415	rs9349415
rs9349416	rs9349416
rs1485780	rs1485780
rs9381579	rs9381579
rs10456570	rs10456570
rs6903331	rs6903331
rs9395286	rs9395286
rs13212790	rs13212790
rs9463342	rs9463342
rs10948367	rs10948367
rs7754971	rs7754971
rs9473123	rs9473123
rs9463335	rs9463335
rs9395285	rs9395285

	rs9369717	rs9369717	
	rs9473128	rs9473128	
	rs9381581	rs9381581	
	rs9296567	rs9296567	
	rs4711878	rs4711878	
	rs9349409	rs9349409	
	rs9369693	rs9369693	
	rs9473122	rs9473122	
	rs9381578	rs9381578	
	rs9349417	rs9349417	
	rs9395262		
	rs2396825		
	rs2171086		
**rs9349407**[2,4]	**rs9349407**	**rs9349407**	**rs9349407**
CD2AP	rs9296559	rs9296559	rs9296559
	rs9296558	rs9296558	rs9296558
	rs9473119	rs9473119	rs9473119
	rs9367279	rs9367279	rs9367279
	rs4715019	rs4715019	rs4715019
	rs1004173	rs1004173	rs1004173
	rs7738044	rs7738044	rs7738044
	rs13211285	rs13211285	rs13211285
	rs4711880	rs4711880	rs4711880
	rs1872505	rs1872505	rs1872505
	rs9473126	rs9473126	rs9473126
	rs4715025	rs4715025	rs4715025
	rs9369716	rs9369716	rs9369716
	rs10948361	rs10948361	
	rs6931478	rs6931478	
	rs9381564	rs9381564	
	rs1931837	rs1931837	
	rs4715018	rs4715018	
	rs9369695	rs9369695	

rs9473117	rs9473117
rs9381562	rs9381562
rs7740963	rs7740963
rs1931833	rs1931833
rs7749271	rs7749271
rs7767350	rs7767350
rs10948363	rs10948363
rs9296561	rs9296561
rs7749167	rs7749167
rs9296564	rs9296564
rs7754282	rs7754282
rs6904764	rs6904764
rs9349413	rs9349413
rs74607583	rs74607583
rs2151974	rs2151974
rs2151975	rs2151975
rs2171089	rs2171089
rs9367284	rs9367284
rs9381575	rs9381575
rs9395279	rs9395279
rs9357546	rs9357546
rs9395283	rs9395283
rs9349415	rs9349415
rs9349416	rs9349416
rs1485780	rs1485780
rs9381579	rs9381579
rs10456570	rs10456570
rs6903331	rs6903331
rs9395286	rs9395286
rs13212790	rs13212790
rs9463342	rs9463342
rs10948367	rs10948367
rs7754971	rs7754971
rs9473123	rs9473123

	rs9463335	rs9463335	
	rs9395285	rs9395285	
	rs9369717	rs9369717	
	rs9473128	rs9473128	
	rs9381581	rs9381581	
	rs9296567	rs9296567	
	rs4711878	rs4711878	
	rs9349409	rs9349409	
	rs9369693	rs9369693	
	rs9473122	rs9473122	
	rs9381578	rs9381578	
	rs9349417	rs9349417	
	rs9395262		
	rs2396825		
	rs2171086		
**rs17817600**[1,2]	**rs17817600**	**rs17817600**	**rs17817600**
PICALM	rs34700169	rs34700169	rs34700169
	rs66802900	rs66802900	rs66802900
	rs71465611	rs71465611	rs71465611
	rs10501602	rs10501602	rs10501602
	rs12791520	rs12791520	rs12791520
	rs117564362	rs117564362	rs117564362
	rs17817648	rs17817648	rs17817648
	rs28365806	rs28365806	rs28365806
	rs17745024	rs17745024	rs17745024
	rs41489645	rs41489645	rs41489645
	rs34675784	rs34675784	rs34675784
	rs67282763	rs67282763	rs67282763
	rs35585561	rs35585561	rs35585561
	rs34223518	rs34223518	rs34223518
	rs12798898	rs12798898	rs12798898
	rs35866111	rs35866111	rs35866111
	rs34874199	rs34874199	rs34874199

rs67719619	rs67719619	rs67719619
rs12804751	rs12804751	rs12804751
rs35220752	rs35220752	rs35220752
rs12787412	rs12787412	rs12787412
rs34959028	rs34959028	rs34959028
rs12795381	rs12795381	rs12795381
rs34766621	rs34766621	rs34766621
rs34003294	rs34003294	rs34003294
rs68116964	rs68116964	rs68116964
rs12788680	rs12788680	rs12788680
rs12787556	rs12787556	rs12787556
rs34332755	rs34332755	rs34332755
rs56191920	rs56191920	rs56191920
rs55932210	rs55932210	rs55932210
rs35992035	rs35992035	rs35992035
rs12798065	rs12798065	rs12798065
rs35519080	rs35519080	rs35519080
rs12795833	rs12795833	rs12795833
rs7118279	rs7118279	rs7118279
rs56094546	rs56094546	rs56094546
rs12805520	rs12805520	rs12805520
rs11821654	rs11821654	rs11821654
rs67682051	rs67682051	rs67682051
rs12798229	rs12798229	rs12798229
rs12788654	rs12788654	rs12788654
rs17817931	rs17817931	rs17817931
rs12802399	rs12802399	rs12802399
rs12790526	rs12790526	rs12790526
rs66494408	rs66494408	rs66494408
rs17817992	rs17817992	rs17817992
rs11234483	rs11234483	
rs17148704	rs17148704	
rs12802064	rs12802064	
rs34584483	rs34584483	

	rs12794211	rs12794211	
	rs12290316		
	rs7107455		
	rs12283410		
	rs34609418		
	rs3213934		
	rs12274987		
	rs12790645		
**rs9271192**[1,2,5,6]	**rs9271192**	**rs9271192**	**rs9271192**
HLA-DRB5/HLA-DRB1			
**rs28834970**[1,2]	**rs28834970**	**rs28834970**	**rs28834970**
PTK2B	rs73223431	rs73223431	
	rs17057043	rs17057043	
	rs6987305	rs6987305	
	rs2322599	rs2322599	
	rs755951		
**rs11218343**	**rs11218343**	**rs11218343**	**rs11218343**
SORL1	rs11218342	rs11218342	rs11218342
	rs720099	rs720099	rs720099
	rs1792124	rs1792124	rs1792124
	rs7131432	rs7131432	rs7131432
	rs3781835	rs3781835	rs3781835
	rs3781838	rs3781838	rs3781838
	rs2276412	rs2276412	rs2276412
	rs77819448	rs77819448	rs77819448
	rs75439772	rs75439772	rs75439772
**rs10498633**	**rs10498633**	**rs10498633**	**rs10498633**
SLC24A4/RIN3	rs12881735	rs12881735	rs12881735
	rs36026988	rs36026988	

**rs8093731**	**rs8093731**	**rs8093731**	**rs8093731**
DSG2	rs73416205	rs73416205	rs73416205
	rs59665004	rs59665004	rs59665004
	rs7229430	rs7229430	rs7229430
	rs75669155	rs75669155	rs75669155
	rs16961997	rs16961997	rs16961997
	rs117748556	rs117748556	rs117748556
	rs116945457	rs116945457	rs116945457
	rs80281026	rs80281026	rs80281026
	rs117247309	rs117247309	rs117247309
	rs117389786	rs117389786	rs117389786
	rs118162451	rs118162451	rs118162451
**rs35349669**	**rs35349669**	**rs35349669**	**rs35349669**
INPP5D	rs28669088	rs28669088	
	rs28534487	rs28534487	
	rs28655385	rs28655385	
	rs28459768	rs28459768	
	rs28478933	rs28478933	
	rs28539971	rs28539971	
	rs35877172	rs35877172	
	rs28605534	rs28605534	
	rs28609111	rs28609111	
	rs7607812	rs7607812	
	rs7559212	rs7559212	
	rs7607736	rs7607736	
	rs28576692	rs28576692	
	rs55801407	rs55801407	
	rs7568027	rs7568027	
**rs190982**	**rs190982**	**rs190982**	**rs190982**
MEF2C	rs304132		
**rs2718058**	**rs2718058**	**rs2718058**	**rs2718058**

NME8	rs4723711		
**rs1476679**	**rs1476679**	**rs1476679**	**rs1476679**
ZCWPW1	rs34919929	rs34919929	rs34919929
	rs34995835	rs34995835	
	rs2405442	rs2405442	
	rs6971558		
	rs12539172		
	rs2906657		
	rs5015756		
**rs10838725**[1,2]	**rs10838725**	**rs10838725**	**rs10838725**
CELF1	rs10838731	rs10838731	rs10838731
	rs10838726	rs10838726	
	rs11039290	rs11039290	
	rs6485758	rs6485758	
	rs7933019	rs7933019	
	rs61895112	rs61895112	
	rs2280231	rs2280231	
	rs11039332	rs11039332	
	rs12577383	rs12577383	
	rs7927445	rs7927445	
	rs7114011	rs7114011	
	rs7131262	rs7131262	
	rs12365079		
	rs7120548		
	rs12223593		
	rs7934481		
	rs11039244		
	rs2293576		
	rs11039284		
	rs12224672		
	rs12361415		
	rs7103835		

	rs12287076		
	rs10769256		
**rs17125944**	**rs17125944**	**rs17125944**	**rs17125944**
FERMT2	rs17125924	rs17125924	rs17125924
**rs7274581**[1,2]	**rs7274581**	**rs7274581**	**rs7274581**
CASS4	rs113902203	rs113902203	
	rs927174	rs927174	
	rs76842328	rs76842328	
	rs79181856		
	rs56012565		
	rs718022		
	rs6014724		
	rs6024870		
	rs6069737		
	rs73156368		

HapMap3 Warnings

1. Query SNP not in HapMap3_r2
2. No matching proxy snps found
3. No LD data is available for rs11767557 in HapMap3_r2, panel CEU
4. No LD data is available for rs9349407 in HapMap3_r2, panel CEU

1000Genomes Warnings

5. Query SNP not in 1000GenomesPilot1
6. No matching proxy SNPs found
7. No LD data available for rs3748140 in 1000GenomesPilot 1, panel CEU
