# Supplementary material for: Connecting the Dots: Potential of Data Integration to Identify Regulatory SNPs in Late-Onset Alzheimer's Disease GWAS Findings
Source: PLoS One. 2014 Apr 17;9(4):e95152. doi: 10.1371/journal.pone.0095152 (PMC3990600; doi:10.1371/journal.pone.0095152)
Supplement: Table S2 — RegulomeDB Scores and Coordinates for all associated SNPs and SNPs in LD r2 ≥0.8. (PDF) [file pone.0095152.s002.pdf]

Table S2. RegulomeDB Scores and Coordinates for all associated SNPs and SNPs in LD $r^2 \geq 0.8$.

Coordinate (0-based)	dbSNP ID	Regulome DB Score
chr11:59936978	rs667897	1b
chr11:47434985	rs2293576	1f
chr11:47461692	rs7103835	1f
chr11:47509136	rs7933019	1f
chr11:47530023	rs6485758	1f
chr11:47572278	rs11039290	1f
chr11:47600437	rs2280231	1f
chr11:47662931	rs7120548	1f
chr11:47811308	rs7114011	1f
chr11:59885119	rs1303615	1f
chr11:59936756	rs617135	1f
chr11:59961485	rs11230180	1f
chr11:59966294	rs2123314	1f
chr11:59989429	rs2081547	1f
chr11:60013856	rs655231	1f
chr15:85425096	rs12917429	1f
chr15:85429355	rs12909280	1f
**chr7:100004445**	**rs1476679**	**1f**
chr8:27220309	rs17057043	1f
chr11:60019149	rs636317	2a
chr11:60019160	rs636341	2a
chr11:85815029	rs1237999	2a
**chr19:1046519**	**rs3764650**	**2a**
chr11:59936925	rs7933202	2b
chr11:85811237	rs542126	2b
**chr12:117295332**	**rs17429217**	**2b**
chr19:1047686	rs4147911	2b
chr20:54997567	rs6024870	2b
chr2:127888336	rs11689287	2b
chr3:64918621	rs812651	2b
chr6:47447040	rs4715019	2b
chr8:27219986	rs73223431	2b
**chr8:27466314**	**rs1532278**	**2b**
chr8:27468502	rs867230	2b
chr11:59927483	rs602396	3a
chr11:59962165	rs7926344	3a
chr2:127847929	rs35114168	3a
**chr6:47452269**	**rs9296559**	**3a**
chr6:47463125	rs9349409	3a
chr6:47480675	rs4711880	3a
chr6:47549494	rs9357546	3a
chr8:27226789	rs755951	3a
chr11:121448253	rs3781835	4

chr11:121460845	rs2276412	4
chr11:121460948	rs77819448	4
chr11:47606864	rs12287076	4
chr11:47789081	rs12223593	4
chr11:59866174	rs502419	4
chr11:59885887	rs564912	4
chr11:59926255	rs107903	4
chr11:59961426	rs10897011	4
chr11:59961785	rs7926729	4
chr11:59962188	rs7926354	4
chr11:60033370	rs7930318	4
chr11:60033447	rs4938932	4
chr11:85630410	rs12805520	4
chr11:85642117	rs35519080	4
chr11:85642443	rs7107455	4
chr11:85652728	rs35220752	4
chr11:85659007	rs35866111	4
chr11:85659260	rs34584483	4
chr11:85676395	rs71465611	4
chr11:85685284	rs17817648	4
chr11:85746036	rs561646	4
chr11:85778750	rs17817992	4
chr11:85811363	rs526904	4
chr11:85828547	rs598726	4
chr11:85828550	rs474479	4
chr14:92938381	rs36026988	4
chr15:85432232	rs34130645	4
chr18:29166725	rs117748556	4
chr19:1047077	rs4147910	4
chr19:1048020	rs73505217	4
chr19:51737990	rs1354106	4
**chr1:207786288**	**rs6701713**	**4**
chr1:207799873	rs10863420	4
chr20:55015165	rs927174	4
chr2:107679235	rs2030674	4
chr2:107679294	rs1916921	4
chr2:234074300	rs7607736	4
chr5:60963674	rs4700424	4
chr5:60974919	rs16893553	4
chr6:33831671	rs9394174	4
chr6:47441870	rs4715018	4
chr6:47451882	rs9296558	4
**chr6:47453377**	**rs9349407**	**4**
chr6:47502023	rs7754282	4
chr8:27466156	rs1532276	4
chr8:27466180	rs1532277	4
chr8:27467820	rs2070926	4

chr9:2722883	rs1026355	4
**chr11:102733162**	**rs12808148**	**5**
**chr11:121435586**	**rs11218343**	**5**
chr11:121441519	rs1792124	5
chr11:121489804	rs75439772	5
chr11:47378395	rs10769256	5
chr11:47500399	rs12224672	5
chr11:47560432	rs61895112	5
chr11:47780753	rs7927445	5
chr11:47883336	rs7934481	5
chr11:59860177	rs502581	5
chr11:59869089	rs580817	5
chr11:59869118	rs580064	5
chr11:59876035	rs512495	5
chr11:59915128	rs558375	5
chr11:59928671	rs11605427	5
chr11:59945744	rs12453	5
chr11:59951432	rs632185	5
chr11:59967525	rs7933805	5
chr11:59970046	rs652303	5
chr11:59970983	rs666555	5
chr11:59975164	rs7928895	5
chr11:59975709	rs673141	5
chr11:59976110	rs7932740	5
chr11:59988861	rs584469	5
chr11:59988905	rs657928	5
chr11:60002934	rs718376	5
chr11:60008150	rs1019671	5
chr11:85625947	rs12798229	5
chr11:85630122	rs11821654	5
chr11:85630836	rs7118279	5
chr11:85641954	rs12283410	5
chr11:85644997	rs12290316	5
chr11:85645036	rs56191920	5
chr11:85645825	rs12788680	5
chr11:85654273	rs67719619	5
chr11:85656901	rs34874199	5
chr11:85659579	rs12798898	5
chr11:85660714	rs34223518	5
chr11:85661220	rs35585561	5
chr11:85662553	rs67282763	5
chr11:85670846	rs12791520	5
chr11:85670944	rs673751	5
chr11:85678533	rs66802900	5
chr11:85756824	rs12802399	5
chr11:85765297	rs493254	5
chr11:85767339	rs12790526	5

chr11:85775963	rs66494408	5
chr11:85776543	rs867611	5
chr11:85787823	rs536841	5
chr11:85788350	rs541458	5
chr11:85795949	rs480781	5
**chr11:85800278**	**rs561655**	**5**
chr11:85820076	rs543293	5
chr11:85856186	rs7110631	5
chr11:85867874	rs10792832	5
**chr14:53400628**	**rs17125944**	**5**
chr14:80200685	rs766649	5
**chr14:92926951**	**rs10498633**	**5**
chr15:85430837	rs3743161	5
**chr15:85430968**	**rs3743162**	**5**
chr15:85431143	rs12442557	5
chr15:85431565	rs34886124	5
chr18:28782790	rs117247309	5
chr18:29065901	rs75669155	5
chr18:29066082	rs7229430	5
**chr18:29088957**	**rs8093731**	**5**
chr18:29192141	rs116945457	5
chr18:29541990	rs118162451	5
**chr18:56752053**	**rs1037757**	**5**
chr19:1048050	rs78410552	5
**chr19:1056491**	**rs3752246**	**5**
chr1:207692048	rs6656401	5
chr1:207786541	rs2093761	5
chr1:207802551	rs4844610	5
chr1:207806729	rs6697005	5
chr20:54998543	rs6014724	5
**chr20:55018259**	**rs7274581**	**5**
chr2:107679473	rs2030675	5
chr2:107684058	rs2971892	5
chr2:107684414	rs2971650	5
chr2:107684956	rs2971894	5
chr2:127888756	rs11680911	5
chr3:64923108	rs704453	5
chr4:155328697	rs10857275	5
chr4:155361273	rs17373598	5
chr5:60947301	rs7728181	5
**chr5:60950210**	**rs11738335**	**5**
chr5:60972282	rs11746049	5
chr5:60975483	rs6896690	5
chr5:60998678	rs11748959	5
chr5:61001388	rs13156637	5
chr5:61003341	rs4699979	5
chr5:61003421	rs4699980	5

chr5:61007720	rs12519720	5
chr5:61007822	rs12519709	5
chr5:61007829	rs12522405	5
chr5:61010842	rs1123764	5
chr5:61013267	rs13154578	5
chr5:88215593	rs304132	5
**chr6:32578529**	**rs9271192**	**5**
**chr6:33825357**	**rs2104362**	**5**
chr6:33826589	rs4713697	5
chr6:47429766	rs9381562	5
chr6:47442376	rs1931837	5
chr6:47445016	rs1004173	5
chr6:47448335	rs9367279	5
chr6:47457708	rs10948361	5
chr6:47461912	rs6931478	5
chr6:47465266	rs9395262	5
chr6:47469272	rs7738044	5
chr6:47480974	rs1872505	5
chr6:47493939	rs7749167	5
chr6:47503496	rs6904764	5
chr6:47505009	rs9473128	5
chr6:47551443	rs9395283	5
chr6:47575331	rs9395286	5
chr7:100091794	rs12539172	5
chr7:143103480	rs56402156	5
chr7:143107875	rs11762262	5
**chr7:143109138**	**rs11767557**	**5**
chr7:99971312	rs2405442	5
**chr8:27195120**	**rs28834970**	**5**
chr8:27211909	rs2322599	5
chr9:2725282	rs1006698	5
chr11:121433792	rs720099	6
chr11:121434427	rs11218342	6
chr11:47466441	rs11039244	6
chr11:47474145	rs12361415	6
**chr11:47557870**	**rs10838725**	**6**
chr11:47568343	rs10838726	6
chr11:47695839	rs11039332	6
chr11:47696595	rs12365079	6
chr11:47836301	rs7131262	6
chr11:59856027	rs1441586	6
chr11:59858711	rs556917	6
chr11:59871603	rs487997	6
chr11:59877696	rs514266	6
chr11:59881523	rs574695	6
chr11:59882305	rs581133	6
chr11:59890673	rs1303621	6

chr11:59899910	rs483629	6
chr11:59901421	rs474123	6
chr11:59906475	rs504272	6
chr11:59908626	rs1786140	6
chr11:59909609	rs493692	6
chr11:59918836	rs684961	6
chr11:59923990	rs652354	6
chr11:59930965	rs664034	6
chr11:59942814	rs7935829	6
chr11:59945064	rs624663	6
chr11:59948373	rs17602572	6
chr11:59964991	rs7116190	6
chr11:59965724	rs11230183	6
chr11:59973928	rs7939882	6
chr11:59974628	rs668287	6
chr11:59977400	rs604085	6
chr11:59979407	rs1285231	6
chr11:59980314	rs11230194	6
chr11:59981768	rs670854	6
chr11:59981800	rs4939320	6
chr11:59982469	rs11603507	6
chr11:59986709	rs688460	6
chr11:59986816	rs56357056	6
chr11:59993345	rs1349667	6
chr11:59996265	rs11601689	6
chr11:59998962	rs2015475	6
chr11:60000573	rs7936120	6
chr11:60001324	rs1426250	6
chr11:60002191	rs678384	6
chr11:60002260	rs1426249	6
chr11:60002261	rs12221613	6
chr11:60005423	rs72920867	6
chr11:60006764	rs650943	6
chr11:60011012	rs611418	6
chr11:60012672	rs603648	6
chr11:60013450	rs600064	6
chr11:60023086	rs1562990	6
chr11:60025564	rs6591559	6
chr11:60026612	rs4938931	6
chr11:60028141	rs7108663	6
chr11:60028939	rs1530914	6
chr11:60029930	rs1026256	6
chr11:60029948	rs1026255	6
chr11:60030456	rs1026254	6
chr11:60030558	rs1026252	6
chr11:60031398	rs10736700	6
chr11:60039916	rs2162254	6

chr11:60041295	rs7107627	6
chr11:85626200	rs12802064	6
chr11:85626737	rs12274987	6
chr11:85627038	rs3213934	6
chr11:85627940	rs34609418	6
chr11:85644666	rs55932210	6
chr11:85648189	rs34003294	6
chr11:85665524	rs41489645	6
chr11:85681388	rs10501602	6
chr11:85686237	rs28365806	6
chr11:85689510	rs34675784	6
chr11:85689711	rs587038	6
chr11:85703748	rs34959028	6
chr11:85703995	rs12794211	6
chr11:85704065	rs12795381	6
chr11:85709603	rs34332755	6
chr11:85710616	rs35992035	6
chr11:85713174	rs12795833	6
chr11:85718230	rs602222	6
chr11:85721331	rs17148704	6
chr11:85724660	rs636355	6
chr11:85740408	rs609903	6
chr11:85814485	rs586274	6
chr14:80189975	rs17764518	6
chr14:80193187	rs72696796	6
chr14:80206974	rs12588956	6
chr14:80228799	rs72698405	6
chr14:80230202	rs12586501	6
chr14:92932827	rs12881735	6
chr18:28823074	rs80281026	6
chr18:29082230	rs59665004	6
chr18:29444479	rs117389786	6
chr19:51731175	rs7245846	6
chr19:51736382	rs33978622	6
chr19:51737120	rs34813869	6
**chr1:207784967**	**rs3818361**	**6**
chr1:207798715	rs10779336	6
chr1:207800388	rs1830763	6
chr1:207804140	rs1408077	6
chr20:54988071	rs73156368	6
**chr2:107678487**	**rs1357692**	**6**
chr2:107678997	rs1916920	6
chr2:107685862	rs1524288	6
**chr2:127889636**	**rs7561528**	**6**
chr2:127894483	rs730482	6
**chr2:232066650**	**rs753855**	**6**
**chr2:234068475**	**rs35349669**	**6**

chr2:234069265	rs28534487	6
chr2:234069511	rs28459768	6
chr2:234071161	rs55801407	6
chr2:234071248	rs7559212	6
chr3:64899399	rs807411	6
chr3:64930074	rs704457	6
chr4:155313307	rs10517591	6
chr4:155314757	rs2130714	6
chr4:155330881	rs990185	6
chr4:155345394	rs35190023	6
**chr4:155347392**	**rs1466662**	**6**
chr4:155349775	rs17301482	6
chr4:155354915	rs4696572	6
chr4:155362530	rs62330361	6
chr4:30473085	rs2175560	6
chr4:30473098	rs1980054	6
chr4:30478872	rs4692459	6
chr4:30479580	rs16867945	6
chr5:60949236	rs7714841	6
chr5:60953098	rs16893320	6
chr5:60956219	rs35438183	6
chr5:60962740	rs35673179	6
chr5:60962765	rs13175381	6
chr5:60962985	rs6883938	6
chr5:60971821	rs7704929	6
chr5:60977097	rs13156487	6
chr5:60990952	rs11740126	6
chr5:60994866	rs11747263	6
chr5:60996141	rs13163936	6
chr5:60997308	rs4700430	6
chr5:60999029	rs12517109	6
chr5:60999359	rs12520360	6
chr5:61003694	rs4700432	6
chr5:61005428	rs13170002	6
chr5:61006197	rs116432267	6
chr5:61010604	rs1123763	6
chr5:61011111	rs13159935	6
chr5:61013775	rs11738462	6
chr6:33830803	rs1555685	6
chr6:33833004	rs3998107	6
chr6:47433750	rs9369693	6
chr6:47440564	rs9369695	6
chr6:47443805	rs9381564	6
chr6:47450617	rs9473119	6
chr6:47456117	rs4711878	6
chr6:47475338	rs9473123	6
chr6:47487761	rs10948363	6

chr6:47494758	rs9296564	6
chr6:47511490	rs9349413	6
chr6:47515629	rs2151974	6
chr6:47515810	rs2171089	6
chr6:47516368	rs9367284	6
chr6:47528763	rs9381575	6
chr6:47542863	rs9395279	6
chr6:47551860	rs9349415	6
chr6:47552179	rs9369716	6
chr6:47553401	rs9296567	6
chr6:47562914	rs6903331	6
chr6:47576366	rs13212790	6
chr6:47585105	rs9463342	6
chr7:100012333	rs34919929	6
chr7:100013456	rs5015756	6
chr7:143099132	rs10808026	6
chr7:37844262	rs4723711	6
chr7:99984088	rs2906657	6
chr7:99990363	rs34995835	6
chr8:27464518	rs11136000	6
chr8:27464928	rs4236673	6
chr9:2742607	rs2034763	6
chr11:121426869	rs7131432	No Data
chr11:121453516	rs3781838	No Data
chr11:47564784	rs11039284	No Data
chr11:47607134	rs10838731	No Data
chr11:47774237	rs12577383	No Data
chr11:59867912	rs574704	No Data
chr11:59870195	rs521952	No Data
chr11:59870787	rs516478	No Data
chr11:59877142	rs555635	No Data
chr11:59878000	rs563803	No Data
chr11:59880037	rs540170	No Data
chr11:59881560	rs574798	No Data
chr11:59903286	rs525794	No Data
chr11:59907096	rs510518	No Data
chr11:59907398	rs534273	No Data
chr11:59911160	rs569046	No Data
chr11:59913805	rs476722	No Data
chr11:59914266	rs595481	No Data
chr11:59914725	rs583296	No Data
chr11:59916748	rs617916	No Data
chr11:59916904	rs606588	No Data
chr11:59918161	rs688030	No Data
chr11:59932578	rs7926954	No Data
**chr11:59939306**	**rs610932**	**No Data**
chr11:59942080	rs634475	No Data

chr11:59942756	rs662196	No Data
chr11:59943108	rs2278867	No Data
chr11:59944579	rs72918674	No Data
chr11:59944809	rs7946992	No Data
chr11:59944897	rs631853	No Data
chr11:59947251	rs583791	No Data
chr11:59957091	rs636147	No Data
chr11:59958379	rs2081545	No Data
chr11:59959842	rs1834550	No Data
chr11:59965788	rs11230184	No Data
**chr11:59971794**	**rs670139**	**No Data**
chr11:59972887	rs599862	No Data
chr11:59973058	rs588084	No Data
chr11:59975077	rs7929589	No Data
chr11:59978254	rs764859	No Data
chr11:59981217	rs1426253	No Data
chr11:59981618	rs603568	No Data
chr11:59984675	rs56273223	No Data
chr11:59986332	rs1820428	No Data
chr11:59986398	rs4492839	No Data
chr11:59990590	rs612738	No Data
chr11:59991332	rs11826180	No Data
chr11:59991846	rs620612	No Data
chr11:59994151	rs673996	No Data
chr11:59996993	rs11230201	No Data
chr11:59997665	rs600550	No Data
chr11:60001572	rs676309	No Data
chr11:60004827	rs621965	No Data
chr11:60005172	rs633463	No Data
chr11:60012153	rs627081	No Data
chr11:60013673	rs654415	No Data
chr11:60014895	rs592894	No Data
chr11:60020111	rs672399	No Data
chr11:60020873	rs675655	No Data
chr11:60021505	rs4939328	No Data
chr11:60021506	rs4939329	No Data
chr11:60021507	rs7396753	No Data
chr11:60024007	rs7121656	No Data
chr11:60027308	rs1365247	No Data
chr11:60028916	rs1530915	No Data
chr11:60030555	rs1026253	No Data
chr11:60031269	rs7128450	No Data
chr11:60031475	rs10736701	No Data
**chr11:60034428**	**rs4938933**	**No Data**
chr11:85624637	rs12790645	No Data
chr11:85643505	rs12798065	No Data
chr11:85644399	rs11234483	No Data

chr11:85645503	rs12787556	No Data
chr11:85647905	rs68116964	No Data
chr11:85670470	rs117564362	No Data
**chr11:85677470**	**rs17817600**	**No Data**
chr11:85677598	rs34700169	No Data
chr11:85686293	rs17745024	No Data
chr11:85691809	rs645299	No Data
chr11:85702053	rs12804751	No Data
chr11:85703092	rs12787412	No Data
chr11:85704233	rs34766621	No Data
chr11:85724122	rs56094546	No Data
chr11:85726293	rs67682051	No Data
chr11:85733107	rs12788654	No Data
chr11:85734009	rs676733	No Data
chr11:85741129	rs682928	No Data
chr11:85754813	rs17817931	No Data
chr11:85757588	rs677909	No Data
chr11:85760027	rs694011	No Data
chr11:85795523	rs565719	No Data
chr11:85798019	rs631639	No Data
chr11:85812209	rs497816	No Data
chr11:85830156	rs567075	No Data
chr11:85831245	rs573167	No Data
chr11:85858537	rs7941541	No Data
chr11:85868639	rs3851179	No Data
chr14:53391679	rs17125924	No Data
chr14:80189373	rs12586263	No Data
chr14:80189442	rs17174723	No Data
chr14:80190555	rs7158292	No Data
chr14:80192861	rs12589656	No Data
chr14:80194005	rs8021767	No Data
chr14:80202531	rs12590550	No Data
chr14:80232187	rs11628399	No Data
chr14:80240220	rs72698407	No Data
chr14:80241164	rs11628403	No Data
**chr14:80242215**	**rs17764668**	**No Data**
**chr16:87111629**	**rs12933233**	**No Data**
chr18:29063229	rs16961997	No Data
chr18:29083561	rs73416205	No Data
chr19:1048115	rs76348507	No Data
**chr19:51727961**	**rs3865444**	**No Data**
chr1:207679306	rs4844600	No Data
chr1:207685785	rs4266886	No Data
chr1:207685964	rs4562624	No Data
chr1:207698043	rs6661489	No Data
chr1:207786827	rs2093760	No Data
chr1:207795319	rs2296160	No Data

chr1:207798237	rs10863418	No Data
chr1:207800554	rs1408078	No Data
chr20:54995698	rs6069737	No Data
chr20:55003464	rs718022	No Data
chr20:55005223	rs56012565	No Data
chr20:55007953	rs79181856	No Data
chr20:55012991	rs76842328	No Data
chr20:55018468	rs113902203	No Data
chr2:107678668	rs1357694	No Data
chr2:107679739	rs2030677	No Data
chr2:107702745	rs7559405	No Data
chr2:107704280	rs13017797	No Data
**chr2:127894614**	**rs744373**	**No Data**
chr2:234068704	rs28669088	No Data
chr2:234069322	rs28655385	No Data
chr2:234069763	rs28478933	No Data
chr2:234070041	rs28539971	No Data
chr2:234070084	rs35877172	No Data
chr2:234070170	rs28605534	No Data
chr2:234070189	rs28609111	No Data
chr2:234070232	rs28576692	No Data
chr2:234071141	rs7607812	No Data
chr2:234071240	rs7568027	No Data
**chr3:64927024**	**rs704454**	**No Data**
chr3:64929027	rs704456	No Data
chr4:155313843	rs9307922	No Data
chr4:155326316	rs1844672	No Data
chr4:155330281	rs1490674	No Data
chr4:155340148	rs13150461	No Data
chr4:155342791	rs9790842	No Data
chr4:155344633	rs12500118	No Data
chr4:155347495	rs1466661	No Data
chr4:155347766	rs35648280	No Data
chr4:155349157	rs17373494	No Data
chr4:155349318	rs62331930	No Data
chr4:155349419	rs57866349	No Data
**chr4:30468165**	**rs6856768**	**No Data**
chr4:30478802	rs35942547	No Data
chr4:30479230	rs16867940	No Data
chr4:30479638	rs6847958	No Data
chr4:30479661	rs16867949	No Data
**chr4:33203139**	**rs10517270**	**No Data**
chr5:60940717	rs6899064	No Data
chr5:60940775	rs6880041	No Data
chr5:60952546	rs11750531	No Data
chr5:60953326	rs16893322	No Data
chr5:60973576	rs11747208	No Data

chr5:60974062	rs11750644	No Data
chr5:60976609	rs1501845	No Data
chr5:60984610	rs11739353	No Data
chr5:60985698	rs13175459	No Data
chr5:60994827	rs11743702	No Data
chr5:60995036	rs13186555	No Data
chr5:60995875	rs6870236	No Data
chr5:61004445	rs17429209	No Data
chr5:61007445	rs13160822	No Data
chr5:61008305	rs6872706	No Data
chr5:61010937	rs12521924	No Data
chr5:61011199	rs12514378	No Data
**chr5:88223419**	**rs190982**	**No Data**
chr6:33834430	rs1535937	No Data
chr6:33835209	rs1555687	No Data
chr6:33837252	rs1555689	No Data
chr6:47427280	rs1931833	No Data
chr6:47427610	rs7740963	No Data
chr6:47431283	rs9473117	No Data
chr6:47474961	rs9473122	No Data
chr6:47479135	rs9463335	No Data
chr6:47479617	rs13211285	No Data
chr6:47481832	rs9473126	No Data
chr6:47483652	rs4715025	No Data
chr6:47485001	rs7749271	No Data
chr6:47485125	rs7767350	No Data
chr6:47488937	rs9296561	No Data
chr6:47514245	rs901186	No Data
chr6:47515662	rs2151975	No Data
chr6:47551937	rs9349416	No Data
chr6:47554176	rs9395285	No Data
chr6:47554467	rs9369717	No Data
chr6:47556629	rs1485780	No Data
chr6:47556633	rs9381578	No Data
chr6:47556679	rs9381579	No Data
chr6:47559450	rs10456570	No Data
chr6:47568695	rs2396825	No Data
chr6:47580656	rs9349417	No Data
chr6:47580694	rs9381581	No Data
chr6:47585614	rs10948367	No Data
chr6:47590103	rs2171086	No Data
chr6:47590475	rs7754971	No Data
chr7:100079856	rs6971558	No Data
chr7:143099106	rs7791765	No Data
chr7:143108840	rs11763230	No Data
**chr7:37841533**	**rs2718058**	**No Data**
chr8:27208125	rs6987305	No Data

chr8:27462480	rs7982	No Data
chr8:27465311	rs11787077	No Data
chr8:27467685	rs9331896	No Data
**chr8:8999018**	**rs3748140**	**No Data**
chr9:2734709	rs10733413	No Data
chr9:2735052	rs7048820	No Data
**chr9:2742770**	**rs2034764**	**No Data**

Bolded SNPs are published and suggestive GWAS SNPs
